# Supplementary material for: Patterns of Intron Gain and Loss in Fungi
Source: PLoS Biol. 2004 Nov 30;2(12):e422. doi: 10.1371/journal.pbio.0020422 (PMC532390; doi:10.1371/journal.pbio.0020422)
Supplement: Table S1 — Also available at http://genes.mit.edu/NielsenEtAl/. (4.3 MB ZIP). [file pbio.0020422.st001.zip › NielsenEtAl/html/675.html]

AN0609.1.NCU06063.1.MG08288.1.FG04333.1


```
 CLUSTAL W (1.82) Multiple Sequence Alignments - Introns Inserted


Sequence 1: NCU06063.1	580 aa
Sequence 2: FG04333.1	575 aa
Sequence 3: MG08288.1	648 aa
Sequence 4: AN0609.1	546 aa
Alignment Length: 677 aa
Number Identitical Residues: 238 aa
Alignment Score (without introns) 12894


MG08288.1 	MAS---IQPATSIEAPAVERERLSILHGPVDPPLVDFTLGELLELQCQQYGTEECVVIPW
NCU06063.1	MSSPALVPTTIEEGAVLPQTQRPSLLHGPTDPPLVDLTLGELLNLQCLHHGTQEALVIPW
FG04333.1 	MAT----EITNVPSAPSRQQQKLSVLEGPVDPPLVDFTLGELLELQTYQHGNQECLVIPW
AN0609.1  	MAGPVRRLQQTLSHLQAPQPQQLSIVYGPTEPELLDVTLGELLTLQSLQYGDYECLVFPW
          	*: .              : :: *:: **.:* *:*.****** **  ::*  *.:*:**

MG08288.1 	TGARWTYNELNQQSCLIARALLSMGVGMGDRVGIMAGNCEQYIAVFFAVARIGGILVILN
NCU06063.1	TGARWTYNELNHQSQLMARALLGLGIRVGDRVGIMAGNCEQYASLFFAATRIGAILVILN
FG04333.1 	TGARWTYNELNQQSSSLAQSLLDMGIGVGDRVAIMAGNCEQYAAVFFAVAKIGAILVILN
AN0609.1  	TGARWTYADLNDEADRVARGMLAMGIKKGDRVGIMAGNCEQYISIFFAAARV--------
          	******* :**.::  :*:.:* :*:  ****.********* ::***.:::        

MG08288.1 	NTYTKTEAQYALGFTD1CKVFFTTRHIGRLDNELLLAELAEQPGSP-------TVVILRG
NCU06063.1	NTYTPTEAQYALEFSD1CKVLFTTKKIGRLDQGPLLAELKMKSKAP-------KVVIIRG
FG04333.1 	NTYTPTEAMYGLKFSD~SKIFFTTPRIGRLDQTQLLQQLENKKTAP-------KVVMLRG
AN0609.1  	---------------D1CRLLFLTPRINKHSLEDVLAELGPHPKASGTSKALEEIVVIRG
          	               * .:::* * :*.: .   :* :*  :  :..::.: . :*::**

MG08288.1 	DSGR-FMTCKDVLHSGIRYSPRSLHEAQLKVLPYNVCNLQFTSGTTGRPKAAMLTHH2NL
NCU06063.1	DSEG-YQTYDQLLSQGFKVSVERLHHMERKVLPHLVCNLQFTSGTTGLPKAAMLTHH2NI
FG04333.1 	DETGKYETYDSLVTAGRRRNHQRLYQAMTKVLPHQVVNLQFTSGTTGLPKAAMLTHH2NL
AN0609.1  	TYKD-FTTYAQVIERGLPLPPNTLPERESQLQTEDVCNLQFTSGSTGNPKAAMLTHQ~YV
          	     : *  .::  *     . * .   :: .  * *******:** ********:  :

MG08288.1 	VNNARYIGDRMRLTPADVLCCPPPLFHCFGLVLGMLATITHGGKVVYPA~EVFDAPATLR
NCU06063.1	VNNARFIGDRMKLGPDDVLCCPPPLFHCFGLVLGLMSVMTHGGKIVYPA~EVFDAPATLK
FG04333.1 	VNNSRFIGDRMRLGPADVLCCPPPLFHCFGLVLGLLAVVTHGGKIVYPA~EVFDIQATLQ
AN0609.1  	PDHQ-----------------PPP------------DPKPNG------S2ETFDPLATLH
          	 ::                  ***               .:*      : *.**  ***:

MG08288.1 	AVQEEGCTALHGVPAMFDSLLALPEAQKLKASDLKLRTGIVAGAPVPRHLMEQMVSRLGM
NCU06063.1	AIVNERCTAVHGVPAMFDSILALPEAKNLKAADLRLRTGIIAGAPVPRYLMELLVSKLGM
FG04333.1 	AISDEQCTAVHGVPAMFDSLFQAKWPENFNCDN--LRTGIIAGAPVPRYLMELLVNRFGM
AN0609.1  	AISDEKCTALHGVPTMFEAILSFDKPPNFDCSN--LRTGIIAGAPVPRPLMKRLFEELNM
          	*: :* ***:****:**::::    . ::.. :  *****:******* **: :...:.*

MG08288.1 	GEFTSSY------------------GLTEASPTCFNAFTDDVVSRRLTTVGRLMPHAHAK
NCU06063.1	TEFTSSY------------------GLTEASPTCFNAHTDDPIDVRLTTVGTLMPHAHAK
FG04333.1 	TEFTSSY------------------GLTEASPTCFNAFTDDSIDTRLTTVGTLMPHAKAK
AN0609.1  	RQYTSSYGISPGHLKPYKARLINCTGLTEASPTCFNALTTDSIETRLQTVGKVMPHAKAK
          	 ::****. :..  .. .:   ..:************ * * :. ** *** :****:**

MG08288.1 	IVDHDGNVVPLGRRGELCIAGYQLQAGYWNNSEKTGEVMVRDENGVLWLKTGDEAVFDAD
NCU06063.1	IVDRDGQIVPIGTKGELCIGGYQLQAGYWNNSEKTNEVMMRDESGMLWLHTGDEAVFNEQ
FG04333.1 	IVDRDGNIVPVGERGELCVGGYQLQAGYWNNSEKTNETMARDASGVLWLHTGDEAVFDEN
AN0609.1  	IIDANGAIVPVGTRGELCMAGYQLTKGYWNNPEKTAETLVTDEEGTVWLKTGDEAVFTPE
          	*:* :* :**:* :****:.****  *****.*** *.:  * .* :**:*******  :

MG08288.1 	GYCSITGRFKDIIIRG~GENIYPLEIEERLVQHPAVATAVVVGLKSERLGEVVGAFLGPA
NCU06063.1	GYCSITGRFKDIIIRG1GENIYPLEIEERLVAHPAVSMAVVVGLKDQHYGEVVGAFLGLE
FG04333.1 	GYCSITGRFKDIIIRG1GENIYPLEIEERLMDHPAITRAIVVGLKNKHYGEVVGAFVELA
AN0609.1  	GYCTITGRFKDIIIRG1GENIYPLEIEERLTAHPSISLSSVIGIQDSKYGEVVGAFIALA
          	***:************ *************  **::: : *:*::..: *******:   

MG08288.1 	DSHGQRSLPDDAEVREWVRAKLGSHKAPSHVFWLGQGGVPSEVPLTGSGK0RYPKSCVNL
NCU06063.1	KGHQP---MSTEEVREWCRRKLGKHKAPTHVFYLGLDGVPSTVPLTGSGK~---------
FG04333.1 	DGHQK---PEFEELKDWCRKRLGGHKSPAHVFWLGHDDVPATVPLTGSGK~---------
AN0609.1  	EGAKR---PSDDELRAWTRETLGRHKAPQHVFVFGEEGVDATVPVTGSGK~---------
          	..       .  *:: * *  ** **:* *** :*  .* : **:*****          

MG08288.1 	TIQPRIFTKHHQPENVLGTRFPAPLPRPGKYGTRIPAADDILQVSECRRIMNPVGSTTSL
NCU06063.1	---VRKFEMAQLGNKLLEE-----------------------------------------
FG04333.1 	---VRKFEMAKLGDDLLRK-----------------------------------------
AN0609.1  	---VRKVDLRKIAAVVLER-----------------------------------------
          	    * .   :    :*                                           

MG08288.1 	SDPHTSVLFDLSDHQNPTSLHS
NCU06063.1	-------------RRKTSKL--
FG04333.1 	-------------QENVSKL--
AN0609.1  	-------------RRNGQVTA-
          	             :.:
```
